# Effect of different fertilization strategies on the yield, quality of Euryales Semen and soil microbial community

**DOI:** 10.3389/fmicb.2023.1310366

**Published:** 2023-11-30

**Authors:** Dishuai Li, Cheng Qu, Xuemei Cheng, Yexing Chen, Hui Yan, Qinan Wu

**Affiliations:** ^1^School of Pharmacy, Nanjing University of Chinese Medicine, Nanjing, China; ^2^Jiangsu Collaborative Innovation Center of Chinese Medicinal Resources Industrialization, Nanjing, China; ^3^National and Local Collaborative Engineering Center of Chinese Medicinal Resources Industrialization and Formulae Innovative Medicine, Nanjing, China

**Keywords:** Euryales Semen, fertilizer, quality, metabolome, bacterial community, fungal community

## Abstract

**Introduction:**

Euryales Semen, a medicinal herb widely utilized in Asia, faces a critical constraint in its production, primarily attributed to fertilizer utilization. Understanding the impact of different fertilization schemes on Euryales Semen (ES) planting and exploring the supporting mechanism are crucial for achieving high yield and sustainable development of the ES planting industry.

**Methods:**

In this study, a field plot experiment was conducted to evaluate the effects of four different fertilization treatments on the yield and quality of ES using morphological characteristics and metabolomic changes. These treatments included a control group and three groups with different organic fertilizer to chemical fertilizer ratios (3:7, 5:5, and 7:3). The results of this study revealed the mechanisms underlying the effect of the different treatments on the yield and quality of Euryales Semen. These insights were achieved through analyses of soil physicochemical properties, soil enzyme activity, and soil microbial structure.

**Results:**

We found that the quality and yield of ES were the best at a ratio of organic fertilizer to chemical fertilizer of 7:3. The optimality of this treatment was reflected in the yield, soil available nitrogen, soil available phosphorus, and soil enzyme activity of ES. This ratio also increased soil microbial diversity, resulting in an increase and decrease in Proteobacteria and Firmicutes abundances, respectively. In addition, linear discriminant analysis showed that Chloroflexi, Gammaproteobacteria, and Hypocreales-incertae-sedis were significantly enriched in the ratio of organic fertilizer to chemical fertilizer of 7:3. Variance partitioning analysis showed that the soil properties, enzyme activities, and their interactions cumulatively can explain 90.80% of the differences in Euryales Semen yield and metabolome. In general, blending organic and chemical fertilizers at a 7:3 ratio can enhance soil fertility, boost Euryales Semen yield and quality, and bring forth conditions that are agriculturally beneficial to microbial (bacteria and fungi) dynamics.

**Discussion:**

This study initially revealed the scientific connotation of the effects of different fertilization patterns on the planting of Euryales Semen and laid a theoretical foundation for the study of green planting patterns of Euryales Semen with high quality and yield.

## 1 Introduction

Euryales Semen (ES), a dry, mature seed kernel of *Euryale ferox* Salisb., is primarily distributed in the southern regions of China and North Bihar, India (Kumar et al., 2018). Medicinally, ES tends to be applied toward the treatment of various ailments (e.g., diabetes and depression) (Ahmed et al., 2015; Huang et al., 2018, 2020). ES starch content reaches more than 70% and is rich in flavonoids and organic acids. Modern pharmacological studies have shown that organic acids and flavonoids have anti-inflammatory, antioxidant properties and therapeutic effects on the nervous, digestive, and reproductive systems (Tian et al., 2022). Moreover, organic acids and flavonoids can be used as quality indicators in ES-related quality control measures.

Research on Euryales Semen (ES) primarily concentrates on the study of its active ingredients and quality assessment, with limited attention given to ES cultivation. Nonetheless, the significance of robust planting practices as the foundation for the Chinese medicine industry cannot be overstated. Under existing planting patterns, ES is frequently included in paddy-upland rotations with wheat, cress, broad beans, lettuce, and ES-wheat rotation system has a wide range of applications. Wheat, as the main food crop, better lends itself better to the promotion and implementation of crop rotation technology. Furthermore, paddy-upland rotations have been proven to enhance soil quality and crop yield, playing a pivotal role in the sustainable development of ES cultivation (Zhou et al., 2014). However, the widespread use of chemical fertilizers for expediting ES cultivation has raised concerns. These practices have primarily contributed to the degradation of cultivated land soils owing to soil acidification, eutrophication, and nutrient uptake limitations, which may negatively affect the ecosystems of the cultivated land, reducing crop yield and quality and ultimately threatening the environment and human health (Calleja-Cervantes et al., 2015; Ahmed et al., 2017). Therefore, developing the development of proper fertilization measures is essential to resolve the current limitations in ES production. The adoption of organic fertilizers as a substitute for chemical fertilizers is recognized as a significant step toward reducing the environmental impact of chemical fertilizer usage. Reportedly, combining chemical and organic fertilizers can improve soil fertility, crop yield, and crop quality (Ning et al., 2022).

Soil enzymes possess high catalytic capacity and participate in the decomposition of organic matter and nutrient cycling in ecosystems. Soil enzymes are closely associated with soil nutrient levels and serve as valuable indicators of soil fertility. Additionally, microorganisms, as fundamental components of soil, contribute to various ecosystem functions, such as regulating plant growth, managing pollutants, and influencing climate (Manjunath et al., 2018). Previous research has shown that combining chemical and organic fertilizers enriched barley rhizosphere microbial communities, leading to improved root-microbe interactions and enhanced resistance to pathogenic bacteria and environmental stress (Guan et al., 2022). However, understanding how different fertilization methods impact the soil microbial environment concerning ES quality and yield remains a complex issue due to the diverse applications of fertilization and the complexity of microbial communities. Therefore, it is necessary to understand changes in soil fertility and microbial environment under different fertilization measures.

Hypotheses for this study include: (1) a high proportion of organic fertilizer would be beneficial to the improvement of ES yield and quality; and (2) the increase in ES yield and quality is linked to improved soil fertility, and it is postulated that this might also result from the enhancement of the microbial environment. To investigate these hypotheses, field plots were established to evaluate the effects of different fertilization methods on ES yield and quality, assessing ES morphology, yield, and metabolomics. Subsequently, the factors influencing changes in ES yield and quality were discussed in terms of soil fertility, soil enzyme activity, and the soil microbial community, providing a scientific basis for optimizing ES green planting techniques and achieving higher ES yields and quality.

## 2 Materials and methods

### 2.1 Experiment materials

Euryale seeds and Zhenmai No.12 wheat seeds used in this research are provided by Huai’an Chuangxing De Euryale Co., LTD. The soil of the experimental site was classified as sandy loam. The basic physicochemical properties of the initial soil (0–20 cm) are as follows: *Pondus hydrogenii* (pH), 8.52; soil organic matter (SOM), 3.77 g/kg; Total nitrogen, 0.04%; Total phosphorus, 0.06%; Total potassium, 1.70%. The organic fertilizer, provided by Huai’an Dahua Biotechnology Co., LTD., was made from the high-temperature fermentation of straw, chicken, and sheep feces (organic matter ≥45%; (N + P_2_O_5_ + K_2_O) ≥ 5%). Chemical fertilizer produced by Zhushang Fertilizer (Qingdao) Co., LTD., including urea (*N* ≥ 17%), calcium and magnesium phosphate (P_2_O_5_ ≥ 17%), and potassium sulfate (K_2_O ≥ 17%).

### 2.2 Experimental design

This study was carried out in an ES planting base (33°29′24”N, 119°22′48″E) located in Cheqiao Town, Huai’an City, Jiangsu Province, China, and the experiment time was from October 2021 to October 2022. The region belonged to the temperate monsoon climate, with an annual average temperature of 14.7°C, a yearly frost-free period of approximately 240 d, an annual average precipitation of nearly 940 mm, and an annual average sunshine duration of 2,130–2,430 h.

The ES-wheat rotation experiment adopted a random block design, and the plot was newly opened in September 2021. This experiment tested four treatment conditions shown in Table 1, and each treatment consisted of three replicate plots (100 m^2^), and each plot was 20 m long, 5 m wide, and 60 cm deep. Adjacent plots were separated by a field ridge with a width of 0.5 m. The area required for planting a single ES plant is 2.2 m × 2.2 m. Winter wheat was sown in October and harvested in early June, while ES was raised in March, colonized in early June, and harvested in late September.

**Table 1:** Experimental treatment and fertilization amount under crop rotation conditions in 2022.

Treatment	Wheat		Euryales Semen	
	Organic fertilizer (kg ha^−1^)	Chemical fertilizer (kg ha^−1^)	Organic fertilizer (kg ha^−1^)	Chemical fertilizer (kg ha^−1^)
CK	0	0	0	0
T1	270.0	630.0	67.2	156.8
T2	450.0	450.0	112.0	112.0
T3	630.0	270.0	156.8	67.2

Before the ES was colonized, one-third of the organic and chemical fertilizers were used as base fertilizers, while the remaining ones were applied as topdressing in early July and early August, respectively.

### 2.3 Agroonomic traits and yield determination of Euryales Semen

After the ES matured, 10 representative plants were selected from each plot for agronomic investigation, which included measuring the ES number of seeds per berry, berry volume, blade area, seed shell thickness, kernel diameter, seed diameter, and hundred-grain weight. Additionally, the crop yield of ES was counted after all the ES had been harvested.

### 2.4 Soil and Euryales Semen sampling

On September 28, 2022, soil sampling was performed using a silt sampler when ES was harvested. Five sub-samples of silt (0–10 cm deep) were collected from the ES root in each plot and mixed to form a composite sample. The sludge was placed in dry ice and transported to the laboratory. All soil samples were divided into three parts; one-third was stored at −80°C for soil DNA extraction and amplification, and two-thirds were air-dried in a cool place and ground into powder. The samples were sieved (0.2 mm) to remove visible non-soil materials and then homogenized to determine soil physicochemical properties and enzyme activities.

During harvest, morphological characteristics of Euryales Semen were recorded, and then it was shelled, dried at 40°C, and ground into powder with subsequent sieving (0.23 mm) for metabolomics analysis.

### 2.5 Soil physicochemical properties analyses

In this study, the soil physicochemical properties analyses were determined according to the reported methods (Shi et al., 2022). Soil pH was determined using a pH meter based on a soil-water ratio of 1:2.5 (*w/v*), while soil total nitrogen (TN) was determined by an automatic nitrogen analyzer. The content of available nitrogen (AN) in the soil was determined by the alkaline hydrolysis diffusion method, and the content of available soil phosphorus (AP) was extracted with sodium bicarbonate and determined by the molybdenum blue method. Additionally, the content of available potassium (AK) in soil was extracted with 1 M ammonium acetate and determined by atomic absorption spectrometry, while SOM content was determined by REDOX titration.

### 2.6 Soil enzyme activity assays

In this study, soil enzyme activity was measured according to previous studies (Li C. et al., 2023). Soil urease activity was determined using the indophenol blue colorimetric method to measure NH_3_-N produced by urease hydrolysis of urea. Sucrase activity in soil was determined using the 3,5-dinitrosalicylic acid colorimetric method, while catalase activity was detected using the hydrogen peroxide colorimetric method. Additionally, soil phosphatase activity was determined by the colorimetric method of disodium benzene phosphate.

### 2.7 Euryales Semen metabolomics analysis

#### 2.7.1 Sample solutions

In this study, the exact 4.00 g medicinal material powder was placed in the 50-mL round-bottom centrifuge tube with 10.0 mL of pure ethanol. The solution was vortexed, subjected to ultrasonic cleaning in an ice water bath for 15 min, and then centrifuged at 4000 rpm for 10 min. The upper liquid was transferred to a clean 50-mL centrifuge tube. The extraction was repeated twice using a 60% ethanol solution for the residues, all solutions were combined, and the solvent was evaporated in a water bath (50°C). The resulting solution was redissolved with 1 mL of 80% methanol solution, and the supernatant was centrifuged (14,000 rpm × 10 min) through 0.22 um organic phase microporous membranes for metabolite analysis.

#### 2.7.2 Experimental equipment and conditions.

AB SCIEX Triple TOF™ 5600, a high-resolution time-of-flight mass spectrometer with AB Sciex DuoSpray™ ion source operating in ESI mode, was used in the study. The instrument was equipped with an ultrafast liquid chromatography analysis system with LC-20ADXR pump and automatic sampler CTO-20 AC, and instrument operation was controlled by Analyst® TF 1.6 software (AB Sciex).

On the basis of previous studies, Euryales Semen metabolomics was determined and moderately modified in this study (Li D. et al., 2023). The chromatographic column used in this study was Waters XBridge™ C_18_ column (5 μm, 4.6 × 250 mm) at 30°C. The flow rate was set at 1 mL/min and the injective volume was 10 μL. The mobile phase consisted of 0.1% formic acid water (A)-acetonitrile (B) with a gradient elution as follow: 0–30 min 5–35% B, 30–35 min 35–70% B, 35–40 min 70–95% B, 40–45 min 95–5% B, 45–50 min 5–5% B.

In this experiment, the mass spectrometer used was an ESI source with a Duospray ion source. The temperature of the ion source was set at 600°C with an injection voltage of −4,500 V and a declustering potential of −100 V. Collision energy was −10 V, 60 Psi for GS1 and 60 Psi for GS2, and 40 Psi for curtain gas (CUR). Negative ion mode was used for measurement, and the MS/MS mass scanning range was *m/z* 50–2000. Additionally, dynamic background subtraction (DBS) was deducted from the results.

#### 2.7.3 Metabolomics data analysis

All data were acquired and analyzed using Analyst® TF 1.6 software (AB SCIEX, USA) and MSDIAL software (ver4.80), respectively. Data were submitted to the Mass Spectrometry Data Analysis software (MSDIAL, ver4.80) with automated peak detection and filtering, peak area integration, peak alignment and smoothing, retention time correction, metabolite annotation, etc. Specific parameters were set: under data collection, the mass tolerance of MS1 and MS2 was set to 0.01 Da and 0.025 Da, the retention time range was set to 100 min, and the fragment collection range for MS1 and MS2 was set to 50–2000 Da; under peak detection parameters, the minimum peak height was set to 80 amplitude, and the mass slice width was set to 0.1 Da; the peak smoothing method was selected as linear weighted shift; the sigma window value was set to 0.5 and the MS/MS abundance cut-off value was set to 0 amplitude; the secondary mass spectrometry database (.msp) used was derived from laboratory preprocessing, mainly from the Mass Bank of North America^1^. In the identification items, 0.01 and 0.05 Da were used for MS1 and MS2 tolerances, respectively; the primary spectra of all data were corrected for retention time to within 0.5 min and an MS1 tolerance of 0.015 Da. Finally, identification results were exported to text (.TXT) format and further filtered by manual screening to remove false positive identifications.

### 2.8 DNA extraction, amplification, and high-throughput sequencing

The soil microorganisms were sequenced according to the previous method, and appropriate modifications were made (Yang et al., 2022). Total microbiome DNA was extracted from microbiome samples from different sources by CTAB method (Hangzhou Zeheng Biotechnology Co., LTD) (Ma et al., 2021). The quality of DNA extraction was detected by agarose gel electrophoresis, and the DNA was quantified by ultraviolet spectrophotometer. Primers specific for the V3–V4 region of the bacterial 16S rRNA genes (341F and 805R) and primers for the internal transcribed spacer region 2 (ITS2) region of the fungal ribosomal RNA genes (fITS7 and ITS4) were selected to amplify bacterial and fungal fragments of suitable size for sequencing. The primer sequences are listed in Supplementary Table S1. The PCR procedure was as follows: initial denaturation at 98°C for 10 min, followed by 35 cycles at 98°C for 10 s, 54°C for 30 s and 72°C for 30 s, and a final extension at 72°C for 10 min. The amplified products of the bacterial 16S rRNA gene and the fungal ITS gene were confirmed by 2% agarose gel electrophoresis, and the AMPure XT beads recycling kit (Beckman, San Jose, CA, USA) was used for recycling. Purified PCR products were evaluated using library quantification kits from Agilent 2,100 bioanalyzer (Agilent, USA) and Illumina (Kapa Biosciences, Woburn, MA, USA). Qualified gradient dilutions of each up-sequencing library were mixed in the appropriate ratio according to the amount of sequencing required and denatured to a single strand using NaOH for up-sequencing; 2 × 250 bp double-end sequencing was performed on a NovaSeq 6,000 sequencer.

### 2.9 Sequence analysis

For the double-ended data obtained by sequencing, the samples were split based on the barcode information, and the splice and barcode sequences were removed after data splicing and filtering. Length filtering and denoising were performed by qiime dada2 denoise-paired calls to DADA2. ASV (feature) feature sequences and ASV (feature) abundance tables were obtained, and singleton ASVs were removed. Alpha diversity analysis and beta diversity analysis were performed based on the obtained ASV (feature) feature sequences and ASV (feature) abundance tables. Species annotation was performed based on the ASV (feature) sequence files using the SILVA (Release 138^2^) database with the NT-16S database, and the ASV (feature) abundance tables were used to annotate each species based on the abundance of each species in each sample was counted according to the ASV (feature) abundance table. Based on the species abundance statistics obtained, an analysis of differences between the comparison groups was performed.

### 2.10 Bioinformatics analysis

Principal co-ordinate analysis (PCoA) based on the calculated euclidean distance using the ‘PCoA’ function of the ‘ade’ package in R v1.7.13^3^. Heat map analysis using the ‘pheatmap’ package in Rstudio^4^. Spearman’s rank correlations were performed at the 95% significance level using IBM SPSS Statistics 26 (IBM Corp, Armonk, NY, USA). Significant discriminant taxa (relative abundance >1%) in different samples were identified using linear discriminant analysis effect size (LEfSe) analysis (Segata et al., 2011), and these biomarkers were particularly enriched under different fertilizer management types (*p* < 0.05, linear discriminant analysis score > 4.0). Redundancy analysis (RDA) and variance partitioning analysis (VPA) was performed using the ‘vegan’ package in R v3.6.3 to explore correlations between microbial community structure at the phylum level and selected conditions.

### 2.11 Statistical analysis

ES morphology, soil chemical properties, and soil enzyme activity between different treatment groups were analyzed, and histograms were plotted using IBM SPSS Statistics 26 and GraphPad Prism (v8.0.2, GraphPad Software, San Diego, CA, USA).

## 3 Results

### 3.1 Effects of different fertilizer treatments on the morphological features and yield of Euryales Semen

**Figure 1:**
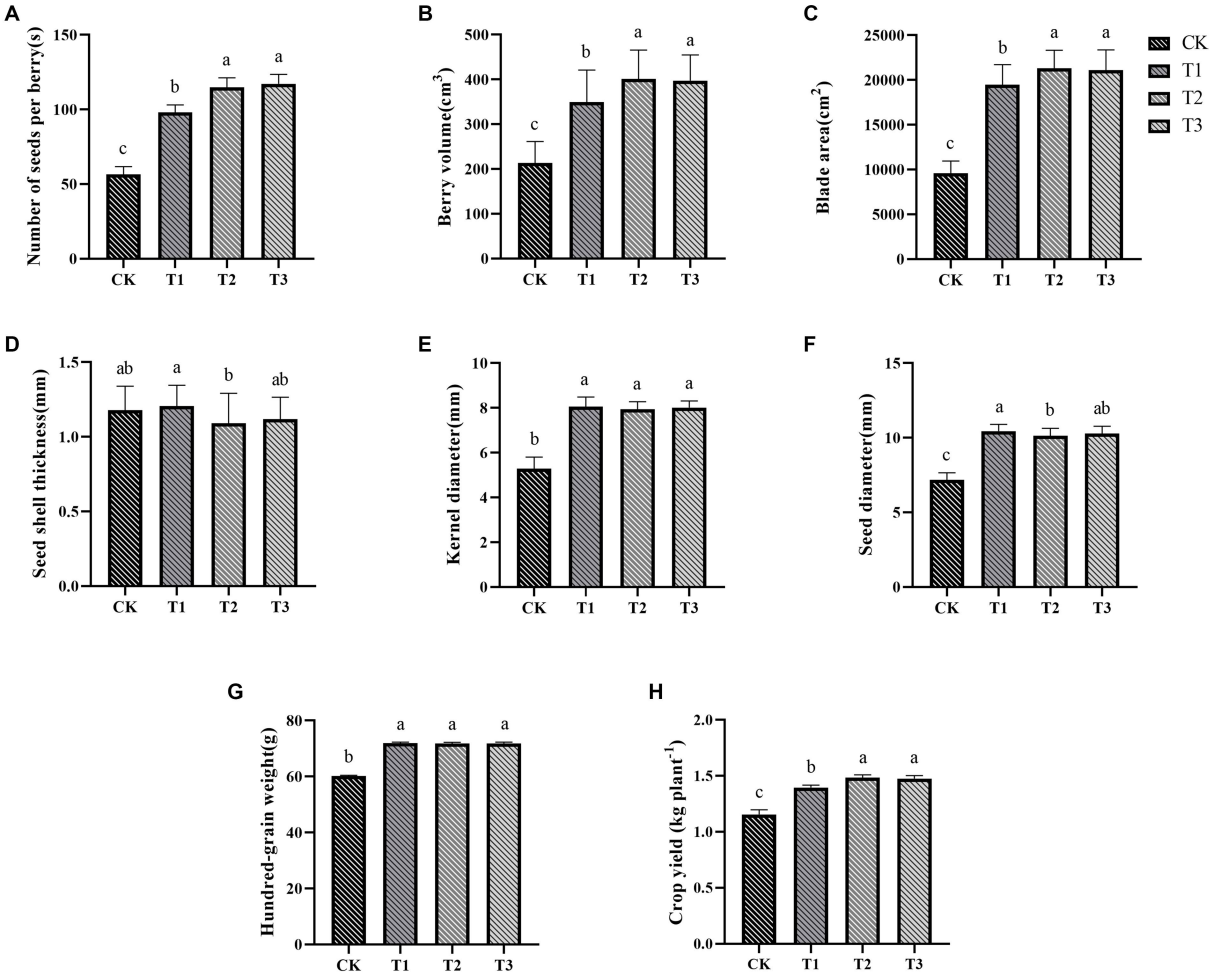
The impact of different fertilization treatments on various parameters of Euryales Semen, including the number of seeds per berry **(A)**, berry volume **(B)**, blade area **(C)**, seed shell thickness **(D)**, kernel diameter **(E)**, seed diameter **(F)**, hundred-grain weight **(G)**, and yield **(H)**. Data are expressed as mean  ±  standard deviation. Values with different lowercase letters indicate significant differences between the four fertilization treatments (*p* <  0.05).

The morphological characteristics of the ES were significantly affected by the different fertilizer treatments (Figure 1). In contrast to the control group, the fertilizer treatments increased the number of seeds per berry, berry volume, blade area, kernel diameter, seed diameter, and hundred-grain weight (Figures 1A–C,E), while seed shell thickness (Figure 1D) remained relatively unaffected. Specifically, the number of seeds per berry, berry volume, and blade area increased with higher organic fertilizer content.

Kernel diameter, seed diameter, seed shell thickness, and hundred-grain weight were less affected by the different fertilizer treatments. The T1 treatment had the largest seed diameter, but it also resulted in the thickest seed shell. Kernel diameter showed no significant differences between the T1, T2, and T3 groups. The use of more organic fertilizer led to thicker ES seed shells and larger seed diameters. It’s commonly believed that larger seed diameter, thinner seed shells, and larger seed kernels make ES more desirable in the market.

The yield of ES crops was significantly impacted by the different fertilization treatments (Figure 1H), and the yield in the fertilization group was higher than that in the control group. The yield of ES showed an upward trend as the amount of organic fertilizer was increased, and the yield of T2 and T3 groups was 5.72–6.21% higher than that of T1 group, but there was no discernible difference in ES yield between the T2 and T3 groups.

### 3.2 Soil physicochemical properties

**Figure 2:**
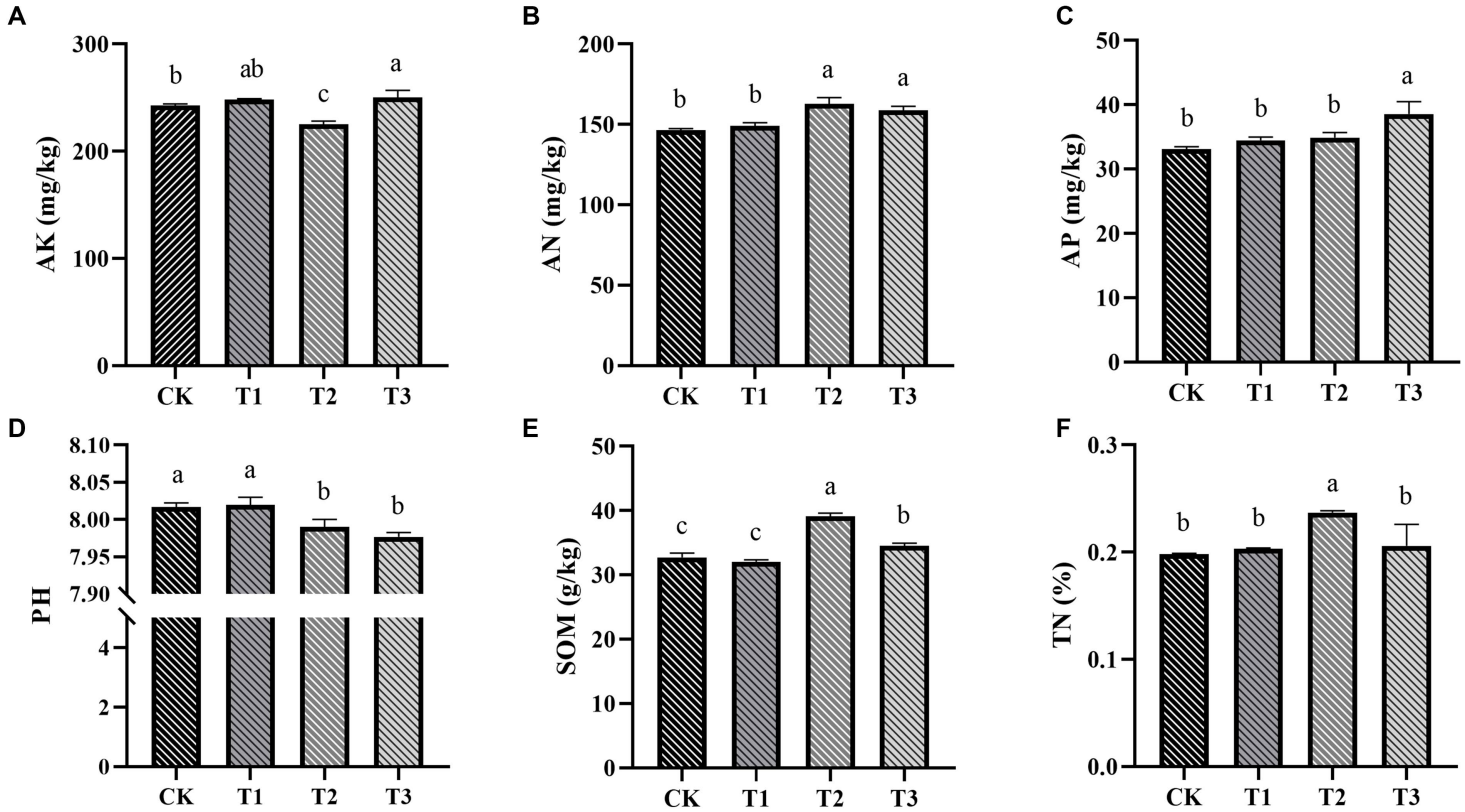
The influence of various fertilization treatments on the soil physicochemical properties in Euryales Semen cultivation, encompassing AK **(A)**, AN **(B)**, AP **(C)**, pH **(D)**, SOM **(E)**, and TN **(F)**. Data are expressed as mean ± standard deviation. Values with different lowercase letters indicate significant differences between the four fertilization treatments (*p* < 0.05).

Fertilizer application significantly influenced soil chemical properties (*p* < 0.05; Figure 2). Specifically, groups T2 and T3 exhibited superior performance, showing higher levels of soil AN, AP, TN, and SOM content. These findings highlight the substantial contribution of strategic organic fertilizer use in enhancing soil fertility. Notably, an increase in the proportion of organic fertilizer application consistently led to a decrease in soil pH (Figure 2D), which has important implications for soil health and nutrient availability. Increasing the utilization of organic fertilizers can effectively boost soil fertility, potentially resulting in higher crop productivity and the promotion of sustainable agricultural practices.

### 3.3 Soil enzyme activity

**Figure 3:**
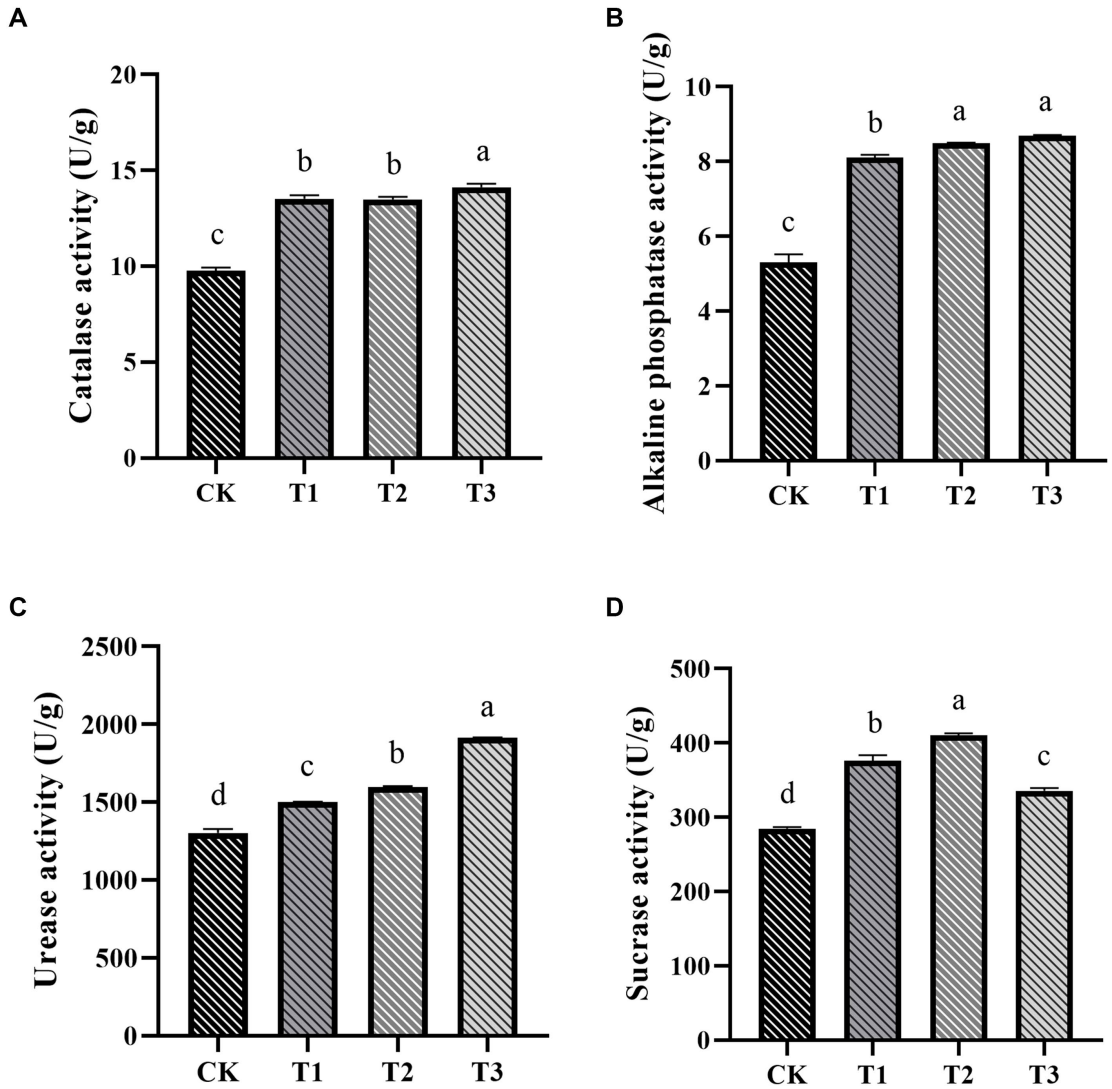
The influence of various fertilization treatments on soil enzyme activities in Euryales Semen cultivation, encompassing soil catalase **(A)**, alkaline phosphatase **(B)**, urease **(C)**, and sucrase activities **(D)**. Data are expressed as mean ± standard deviation. Values with different lowercase letters indicate significant differences between the four fertilization treatments (*p* < 0.05).

The impact of fertilizer application on soil enzyme activity in ES soil was significant (Figure 3). Soil urease, catalase, and alkaline phosphatase activities notably increased with higher organic fertilizer application (Figures 3A–C). In the T3 group, these activities increased by 46.72, 44.35, and 63.96%, respectively, compared to the CK group. Additionally, the T2 group exhibited the highest sucrase activity (Figure 3D). Overall, soil enzyme activity exhibited a positive trend with increasing organic fertilizer application, consistent with previous research (Song D. et al., 2022).

### 3.4 Euryales Semen metabolomics

**Figure 4:**
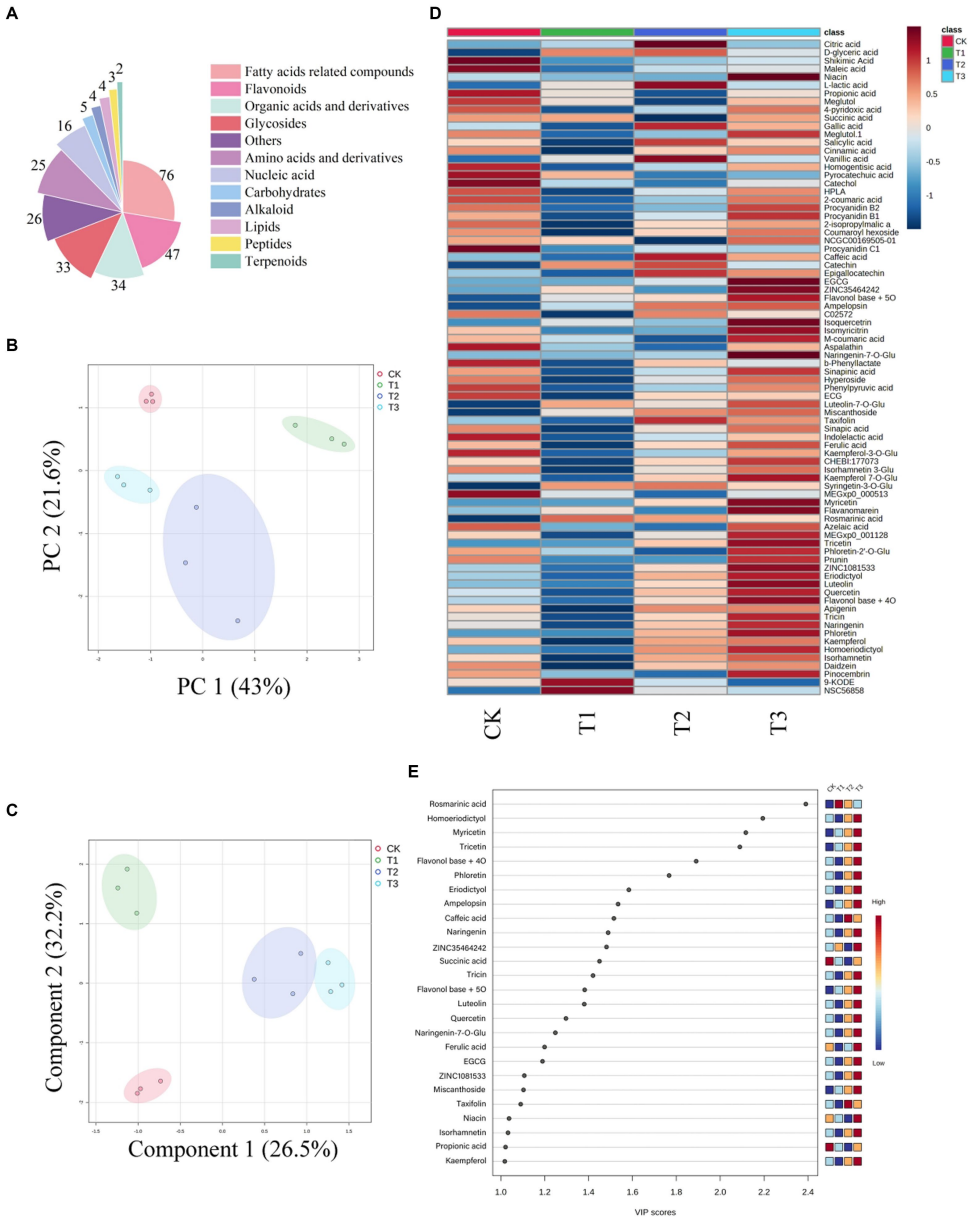
Visualization of metabolic data of Euryales Semen in different treatment groups. Pie chart depicting the biochemical classifications of the 275 identified compounds in Euryales Semen **(A)**. Heatmap showing relative peak area of annotated flavonoids and organic acids **(B)**. Principal component analysis analysis of metabolic differences among different fertilization treatment groups **(C)**. The partial least squares discriminant analysis of metabolism between different fertilization groups **(D)**. Projection of the importance of variables **(E)**.

In this study, we identified 275 compounds, including 76 fatty acids and derivatives, 47 flavonoids and derivatives, and 34 organic acid metabolites and derivatives (Figure 4A). Visualizing the compound peak area thermograms (Figure 4B) revealed significant quality differences in ES among the fertilization treatments. The peak areas of organic acids and flavonoid components were highest in T3, followed by CK and T2, with the lowest in T1. Principal component analysis (PCA) illustrated substantial metabolic variations among the fertilizer treatment groups (Figure 4C). Further, partial least squares discriminant analysis (PLS-DA) (Figure 4D) showed separation between these groups. We identified 26 differential compounds with a variable influence on projection (VIP) > 1 (Figure 4E), focusing on the top 10 VIP values for the subsequent analysis. Our results indicated that increasing organic fertilizer application led to higher peak areas of organic acids and flavonoid components in ES. This evaluation system indicates that an increase in organic fertilizer application will improve ES quality and enhance its medicinal value.

### 3.5 Microbial taxonomical distribution

#### 3.5.1 DNA extraction and sequencing

After removing unwanted sequences and low-quality reads, we had 319,928 high-quality bacterial 16S rRNA gene sequences for analysis. For fungal ITS sequence analysis, we retained 370,049 high-quality sequences. These sequences underwent filtering, de-duplication, and chimera removal to obtain precise Amplicon Sequence Variant signature lists and sequences. The ASV signature sequences for both bacteria and fungi were used for diversity analysis, species identification, and differential analysis.

#### 3.5.2 Alpha and beta diversity analysis

Alpha diversity indices, including Chao 1, Shannon index, Simpson index, and evenness, were significantly impacted by various fertilization treatments (Table 2). According to the study results, from a bacterial and fungal perspective, the Chao 1, Shannon index, Simpson index, and evenness were higher in the T3 group than those in all the other treatment groups. The Chao 1, Shannon index, Simpson index, and evenness were lower in the T2 group than those in all other treatment groups. Overall, the results indicated that high levels of organic fertilizer positively influenced the alpha diversity index, whereas applying the same amount of organic fertilizer as chemical fertilizer had a less favorable effect.

**Table 2:** Effect of different fertilizer treatments on the alpha diversity index of Euryales Semen.

Species	Treatments	Chao 1 index	Simpson index	Shannon index	Evenness
Bacteria	CK	2917.393 ± 96.431a	0.998 ± 0.000a	10.610 ± 0.049b	0.922 ± 0.003b
	T1	2926.178 ± 63.553a	0.998 ± 0.000a	10.576 ± 0.044b	0.9187 ± 0.002b
	T2	2678.752 ± 151.503b	0.995 ± 0.001a	10.230 ± 0.120c	0.899 ± 0.004c
	T3	3127.415 ± 139.244a	0.999 ± 0.000a	10.797 ± 0.070a	0.930 ± 0.003a
Fungi	CK	776.849 ± 34.774a	0.9847 ± 0.001a	7.371 ± 0.053a	0.768 ± 0.007a
	T1	874.390 ± 26.015a	0.985 ± 0.002a	7.469 ± 0.054a	0.765 ± 0.006a
	T2	912.077 ± 67.930a	0.977 ± 0.009a	7.072 ± 0.212b	0.720 ± 0.026b
	T3	911.138 ± 127.112a	0.986 ± 0.001a	7.582 ± 0.159a	0.772 ± 0.017a

Different letter superscripts in the same column of the same species indicate significant differences between treatments (*P* < 0.05).

PCoA was used to compare the differences in the composition of bacterial and fungal communities in the soils of the different fertilizer treatments (Supplementary Figure S1). The results indicated that most of the differences in the composition of the soil microbial communities between the different fertilizer treatment groups were significant. Notably, the fungal compositions of T2 and T3 were similar. PCoA1 and PCoA2 explained 71.29 and 61.97% of the variation in bacterial and fungal community data, respectively, signifying that different fertilizer treatments substantially altered soil bacterial and fungal community composition.

#### 3.5.3 Relative abundance of microbial community

**Figure 5:**
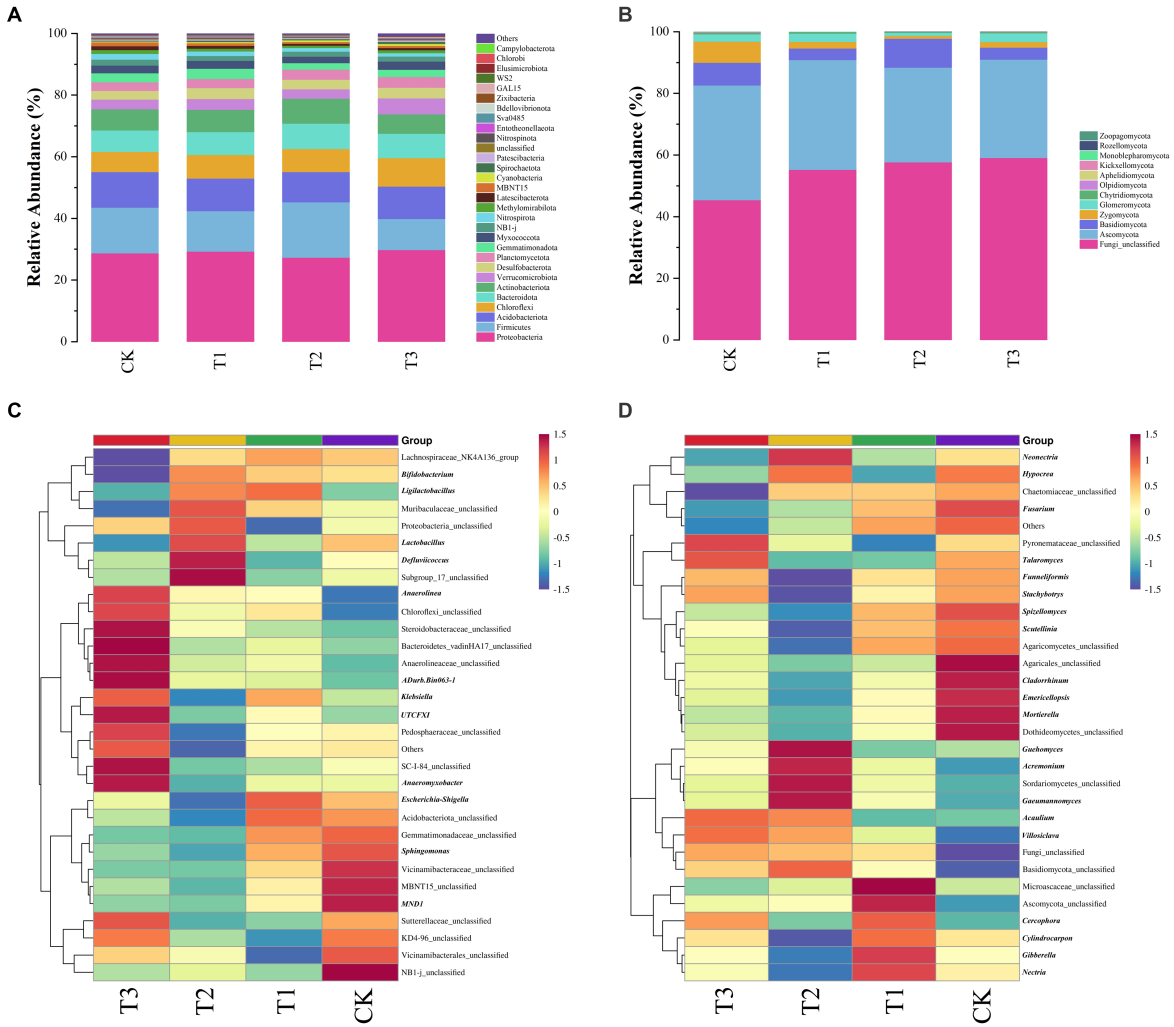
Relative abundance of the main bacterial **(A)** and fungal **(B)** phyla in soil under different fertilizer treatments. Heatmap of the first 31 bacterial genera **(C)** and 31 fungal genera **(D)** in soil under different fertiliser treatments.

The various fertilizer treatments varied the predominant taxa associated with the microbial communities (Figures 5A,B). The 12 fungal phyla and 64 bacterial phyla were measured in all soil samples, and the top 30 species were selected for display based on their abundance.

The dominant bacterial phylum was found to be Proteobacteria with a relative abundance of 27.33–29.78%, followed by Firmicutes (10.06–17.98%), Acidobacteriota (9.78–11.5%), Chloroflexi (6.57–9.23%), Bacteroidota (6.98–8.22%), Actinobacteriota (6.30–8.11%), and Verrucomicrobiota (3.05–5.17%; Figure 5A). In each soil sample, the predominant clade accounted for more than 78.63% of the bacterial 16S rRNA gene sequences. In the T3 treatment, Proteobacteria had the highest relative abundance, whereas Firmicutes had the lowest. In contrast, in the T2 treatment, Proteobacteria had the lowest relative abundance, whereas Firmicutes had the highest.

Among the classified fungal phyla, Unclassified (42.73–63.03%), Ascomycota (30.14–41.05%), Basidiomycota (2.39–18.78%), Zygomycota (0.58–6.82%) and Glomeromycota (0.1–7.68%) were the dominant taxa in the samples from the different fertilizer treatments, accounting for at least 99.06% of all fungal sequences in Figure 5B. Ascomycota had the lowest relative abundance in T2 group, but Basidiomycota had the highest.

#### 3.5.4 Heatmap of bacterial and fungal genera

The heat map revealed a close correlation between fertilizer application conditions and significant differences in soil bacterial and fungal community structure at the genus level (Figures 5C,D).

The 31 most abundant bacterial genera were Vicinamibacterales_unclassified, *Defluviicoccus*, Sutterellaceae_unclassified, Others, Vicinamibacteraceae_unclassified, NB1-j_unclassified_unclassified, SC-I-84_unclassified, Gemmatimonadaceae_unclassified, Steroidobacteraceae_unclassified, and Subgroup_17_unclassified, which was present as an abundant group in all soil samples (abundance >1%). Compared with the other treatments, *Anaerolinea*, Chloroflexi_unclassified, Steroidobacteraceae_unclassified, Bacteroidetes_vadinHA17_unclassified, Anaerolineaceae_unclassified, *ADurb.Bin063-1*, *Klebsiella*, *UTCFX1*, Pedosphaeraceae_unclassified, Others, SC-I-84_unclassified, and *Anaeromyxobacter* were particularly enriched in the T3 treatment, whereas Lachnospiraceae_NK4A136_group, *Bifidobacterium*, Muribaculaceae_unclassified and *Lactobacillus* were decreased (Figure 5C).

Among the 31 most abundant fungal genera, Microascaceae_unclassified, Fungi_unclassified, Others, Ascomycota_unclassified, *Fusarium*, *Guehomyces*, and *Neonectria* were abundant groups in all soil samples (abundance >1%). Compared to the other treatment groups, Pyronemataceae_unclassified, *Talaromyces*, *Acaulium*, and *Villosiclava* were enriched in the T3 treatment, whereas *Neonectria*, Chaetomiaceae_unclassified, *Fusarium*, Other, and Microascaceae_unclassified were reduced (Figure 5D).

#### 3.5.5 Biomarkers associated with different fertilization conditions

**Figure 6:**
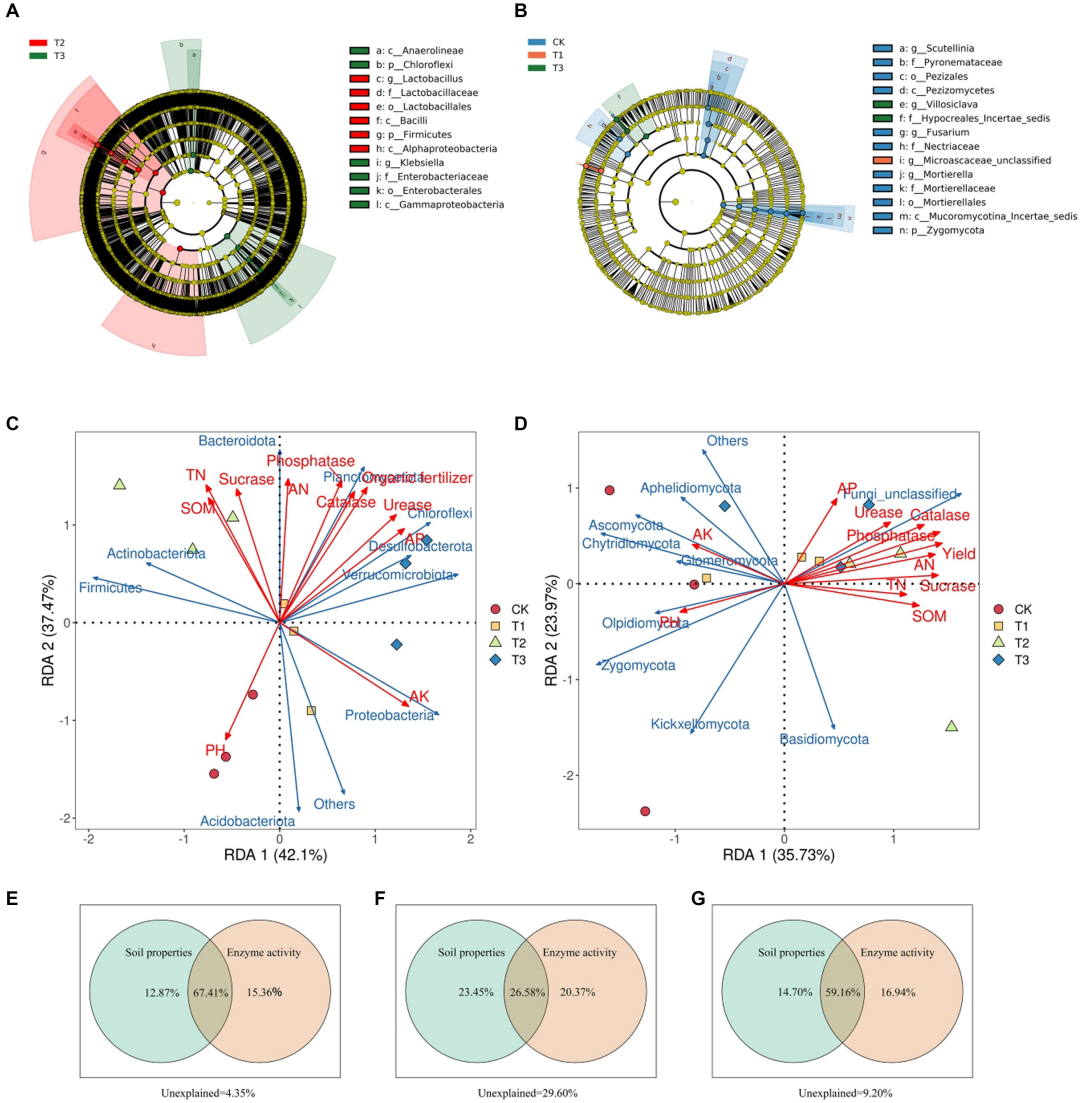
Taxonomic branching diagram of significant enrichment of biomarkers under different treatments based on linear discriminant analysis (LDA) effect size analysis (**A**, bacteria; **B**, fungi). Redundancy analysis of the relationships between microbial community structure, Euryales Semen yield, soil properties, and soil enzyme activity (**C**, bacteria; **D**, fungi). Variance allocation analysis of the effects of soil properties and soil enzyme activity on microbial community structure and Euryales Semen yield and quality **(E–G)**. In the **(A,B)**, LDA effect size analysis was performed to identify the indicator taxa representing each group, and the values were significant (*p* < 0.05) when the LDA score was greater than 4. There are seven rings in the cladogram that represent the Kingdom, phylum, class, order, family, genus, and species from inside to outside, respectively. The different color nodes on the ring represent significant changes in taxonomic composition due to the treatments. Abbreviations for classification levels: K, kingdom; P, phylum; C class; O, order; F, family; G, genus; S, species.

A taxonomic branching diagram based on the LEfSe results displays highly abundant soil microbial communities in each fertilizer treatment group in Figure 6 (*p* < 0.05). LEfSe analysis of the bacterial community (Figure 6A) revealed that Firmicutes and Alphaproteobacteria were significantly enriched in the T2 group, whereas Chloroflexi and Gammaproteobacteria were significantly enriched in T3 group. LEfSe analysis of the fungal communities (Figure 6B) showed that Pezizomycetes, Nectriaceae, and Zygomycota were significantly enriched in the CK group, Microascaceae_unclassified was considerably enriched in the T1 group, and Hypocreales-incertae-sedis was significantly enriched in the T3 group. These findings illustrate that different fertilizer treatments attract distinct soil microorganisms, underscoring the substantial impact of fertilization methods on soil microbial structure.

### 3.6 Effects of soil environmental factors on microbial composition and diversity and Euryales Semen yield and quality

The RDA of microbial community data (relative abundance of phylum-level taxa top 10) and environmental factors (ES yield and soil properties) showed how soil ecological variables affected the structure of soil microbial communities in Figures 6C,D. For bacteria, the first two RDA axes explained 79.57% of the total community variance, and distinct separation was observed among the treatment groups. Acidobacteriota and Others were the most abundant in the CK treatment, and there was a positive correlation between their relative abundance and soil pH and AK. Proteobacteria, Verrucomicrobiota, Desulfobacterota, Chloroflexi, and Planctomycetota were the most abundant in the T3 treated group, with positive correlations with yield, AN, AK, AP, urease activity, catalase activity, and phosphatase activity. Regarding fungi, the first two RDA axes accounted for 59.70% of the total community variance. Others, Aphelidiomycota, Ascomycota, Chytridiomycota, Glomeromycota, Olpidiomycota, Kickxellomycota, and Zygomycota, were the most abundant in the CK treatment, AK and soil pH were both positively correlated with their relative abundances. The unclassified abundance of fungi was positively correlated with AP, urease activity, catalase activity, phosphatase activity, yield, AN, sucrase activity, TN, and SOM.

RDA examines the relative abundance of bacteria and fungi in various treatments, revealing unique patterns for each group. To demonstrate how soil properties, enzyme activities, and their interactions collectively account for variations in soil microbial communities (bacteria and fungi) and their effects on ES production and metabolome, we employ Variation Partitioning Analysis (VPA). Soil properties, enzyme activities, and their interactions cumulatively explained 95.65% of the variation in soil bacterial communities (Figure 6E) and 70.40% of the variation in soil fungal communities (Figure 6F). Soil properties and enzyme activity explained more of the variation in bacterial communities than that in fungal communities. Overall, soil properties, enzyme activity, and their interactions cumulatively explained 90.80% of the outcomes related to ES production and metabolome in Figure 6G. This underscores the comprehensive role of soil properties and enzyme activities in shaping soil microbial structure and influencing ES production and quality.

**Figure 7:**
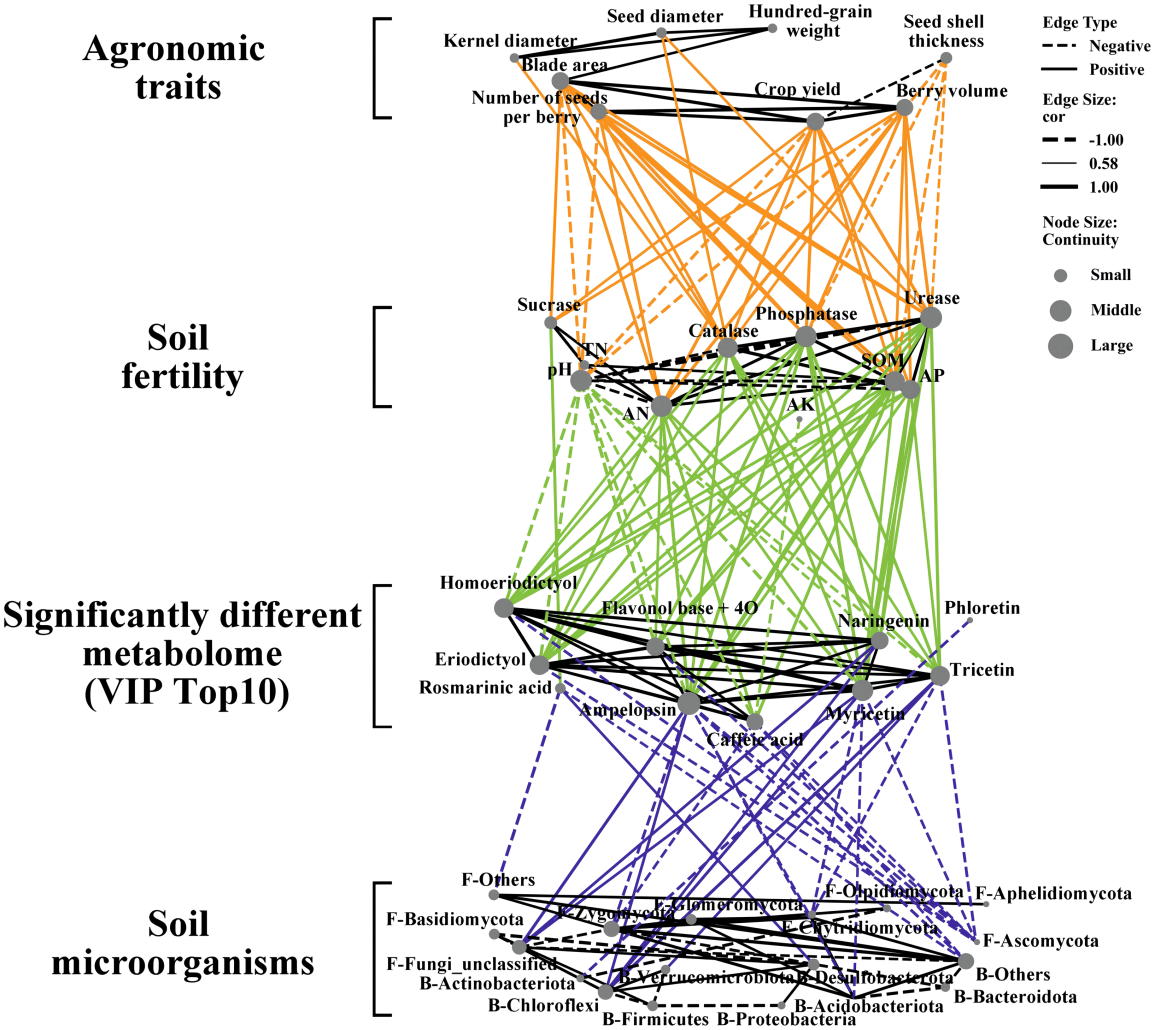
Correlation network between agronomic traits, soil fertility, soil differential metabolites (Top 10 VIP value) and microorganisms (Top 8 bacterial and fungal abundance) between different treatment groups (Spearman, *p* < 0.05). The solid line represents a positive correlation and the dashed line represents a negative correlation.

To better understand the regulatory relationships between ES agricultural traits, soil fertility indicators, soil microbial composition, and metabolite levels, we created a four-tiered correlation network (Figure 7). The correlation analysis of agricultural traits and soil fertility showed a negative correlation between seed shell thickness and soil fertility indicators, aligning with the belief that thinner seed shells indicate higher Euryales Semen quality. With improved soil fertility, we observed the most significant enhancements in blade area, number of seeds, and berry volume. In the correlation analysis between soil fertility and differential metabolites, indicators such as AN, SOM, AP, catalase activity, phosphatase activity, and urease activity played a more pronounced regulatory role in the content of differential metabolites. Fertility indicators consistently affect the levels of differential metabolites, and an increase in these indicators generally leads to higher levels of differential compounds. The levels of various flavonoids and organic acids are generally significantly positively correlated, indicating a consistent influence of the external environment on their contents. When correlating differential metabolites with microbial abundance (i.e., specifically, the top 8 bacteria and top 8 fungi), Ascomycota and Chytridiomycota displayed a significant negative correlation with multiple differential compounds, whereas Chloroflexi exhibited a significant positive correlation with ampelopsin, Naringenin, Myricetin, and tricetin. There is a significant positive correlation between Desulrobacterota and Rosmarinic acid, as well as between Verrucomicrobrota and Tricetin. This may be associated with their roles in sulfur and organic matter recycling. Then, we further analyzed the correlation between microbial abundance and Agronomic traits of ES (Supplementary Figure S2). Bacteroidota, Chloroflexi, and Desulfobacterota are of significant positive correlation with Agronomic traits of ES. Zygomycota, Ascomycota, Acidobacteriota and Chytridiomycota have significant negative correlation with Agronomic traits of ES. This suggests that Chloroflexi may play a significant role in regulating the quality and yield of ES.

## 4 Discussion

### 4.1 Euryales Semen yield and quality are affected by different fertilization conditions

Previous studies have shown that proper substitution of chemical fertilizers with organic fertilizers can improve soil fertility and achieve high yields of wheat and maize (Ning et al., 2022). This practice has also been found to increase the population of interroot microorganisms, promote interactions between roots and microorganisms, and enhance resistance to pathogens and environmental stresses (Guan et al., 2022). Based on the findings of our field trials, a higher proportion of organic fertilizer can increase the yield and quality of ES. The T2 and T3 groups had similar yields, but the quality of ES in the T3 treatment surpassed that of the T2 group, indicating the suitability of the T3 treatment for ES cultivation. Although an adequate supply of fertilizer can promote the growth of ES, increase yield, and enhance quality, excessive fertilizer application may lead to a substantial proliferation of algae in the experimental field. Algae can compete with ES for living space, thus negatively impacting their growth. Therefore, it is essential to carefully regulate the amount of fertilizer applied.

Correlation analysis revealed that soil properties significantly influenced ES morphology and yield, consistent with the findings of Ye et al. (2022), who reported that organic fertilizer effectively enhanced the growth, yield, Vc, and total flavonoid content of pear dates on the Loess Plateau. Similarly, Sousa et al. (2008) showed that the application of organic fertilizer reduced the environmental pollution and increased the content of flavonoids and organic acids in cabbage. Our metabolomic analysis revealed that the T3 group had the highest flavonoid and organic acid contents in ES, reflecting the positive impact of increased organic fertilizer on ES quality.

The combined application of organic and inorganic fertilizers at a reasonable ratio positively affects soil quality and productivity (Elisée Ouédraogo et al., 2007; Kaewpradit et al., 2009). Our experimental data indicated that the T2 and T3 groups significantly improved soil properties compared to the CK group, with advantages in AN, AP, TN, and SOM contents. The nutrient content of chemical fertilizers and organic fertilizers, in terms of N, P, and K, differs significantly. Specifically, despite the high N, P, and K content in chemical fertilizers, their use is always linked to a risk of nutrient loss during ES cultivation. One of the primary strengths of organic fertilizers is that they have an inherently slow nutrient release rate and strong affinity for soil particles, which are advantageous qualities for ES cultivation. As a result, the T2 and T3 groups benefit from improved soil nutrient levels. This indicates that organic fertilizers have great potential to reduce chemical fertilizer use and increase crop yields and are beneficial to the stability and sustainable development of soil ecosystems (Li X. et al., 2022).

### 4.2 Regulation of soil microbial environment under different fertilization conditions

Soil microbes play an important role in the soil and crop ecosystem (D’Acunto et al., 2018; Zhang et al., 2023). The decline in soil microbial diversity has become a global concern, as it plays a vital role in maintaining plant productivity, and a reduction in diversity can compromise critical ecosystem functions and crop yields (Chen et al., 2020). In the present study, the different groups of soil microbial communities (CK, T1, T2, and T3) could be clearly distinguished in the PCoA, indicating that different fertilization conditions significantly affected the soil microbial community composition in the ES-wheat rotation systems. The alpha diversity index of the bacteria was higher in the T3 group than that in the other groups. Various fertilization treatments did not exhibit obvious regular changes on the alpha diversity index of fungi. Fertilizer application can increase soil nutrient concentrations, promoting the rapid growth of bacteria that generally have a higher nutrient demand. In contrast, fungi usually have longer lifecycles and are less reliant on fast growth and reproduction, making them less vulnerable to disruptions caused by excessive nutrients.

The microbial community composition was also influenced by fertilizer application. Among the bacteria, Proteobacteria, Firmicutes, Acidobacteriota, and Chloroflexi were the dominant phylum, accounting for 58.51–62.39% of the bacterial phylum in the soil samples. Proteobacteria are responsible for atmospheric nitrogen fixation in terrestrial ecosystems (Graham and Vance, 2003; Raymond et al., 2004), while Firmicutes and Acidobacteriota are prevalent in various soil environments and may play a role in suppressing fungal diseases in crops (Zhou et al., 2019; Li Y. et al., 2022). Among the fungi, Unclassified, Ascomycota, Basidiomycota, and Zygomycota were the dominant phylum, accounting for 97.71–99.59% of the fungal phylum in the soil samples. Members of Ascomycota exhibit increased diversity after simulated warming, affecting soil carbon cycling processes (Barrera et al., 2019), while Basidiomycota is a major contributor to ecosystem functioning and lignin degradation (Taylor et al., 2015). These phyla may be associated with potential fungal pathogen infections (Kwaśna et al., 2021; Goodwin, 2022).

In our study, the T3 treatment exhibited the highest relative abundance of Proteobacteria, consistent with the findings of Iqbal et al. (2022), who reported higher Proteobacteria abundance in response to high organic fertilizer rates.

At the genus level of bacteria, *Anaerolinea*, Chloroflexi_unclassified, Steroidobacteraceae_unclassified, Bacteroidetes_vadinHA17_unclassified, Anaerolineaceae_unclassified, *ADurb.Bin063-1*, *Klebsiella*, *UTCFX1*, Pedosphaeraceae_unclassified, Others, SC-I-84_unclassified, and *Anaeromyxobacter* were enriched in T3 treatment. *Anaerolinea* is more abundant in high organic fertilizers, where it is able to degrade carbohydrates under anaerobic conditions, participate in the cycling of C, N, S, and F in the soil, and interact with other bacteria to promote soil fertility (Zhang et al., 2019). *Klebsiella* is chemoorganotrophic and has both respiratory and fermentative metabolisms (Vanhooren et al., 1999). At the genus level of fungi, Pyronemataceae_unclassified, *Talaromyces*, *Acaulium*, and *Villosiclava* were enriched in the T3 treatment. The plant-friendly genus, *Talaromyces*, was enriched at the interroot level by organic fertilization (Sommermann et al., 2022), and *Acaulium* abundance was found to increase significantly after organic composting (He et al., 2022). *Acaulium*, which significantly increased after organic composting, has been linked to crop rot symptoms (Tian et al., 2021).

### 4.3 Soil microbial community may impact both the yield and quality of Euryales Semen

Soil properties can influence the structure of soil microbial communities to varying degrees (Ablimit et al., 2022; Zhang et al., 2022). Based on RDA, the relationship between soil microbial community structure (phylum level) and soil properties was evaluated. The effects of soil properties on bacterial and fungal phyla were two-fold. SOM, AN, and AP values were the main factors influencing the bacterial or fungal community structure. The abundance of Actinobacteria and Firmicutes was positively correlated with soil SOM values, which is consistent with experimental results on the effect of bioorganic fertilizer on banana yield and quality (Li Z. et al., 2021). Additionally, Song Y. et al. (2022) reported the close relationship between Acidobacteria and Proteobacteria with crucial soil nutrients like available potassium (AK), available phosphorus (AP), and ammonium nitrogen (AN).

The relationship between microbial taxon abundance at the phylum level, Euryales Semen (ES) metabolome data, and soil properties further supports the impact of soil properties on the ES microbial community structure. ES quality was also significantly correlated with microbial taxon abundance. Li Y. et al. (2022) discovered that organic fertilizers improved soil physicochemical properties, especially the content of total carbon, effective potassium, and effective phosphorus, and improved microorganisms, thus enhancing the quality and yield of *Perilla frutescens*.

Microorganisms have great potential to improve soil quality and fertility through biomineralization and synergistic co-evolution (Gouda et al., 2018). In our study, certain bacteria, including Chloroflexi and Gammaproteobacteria, served as biomarkers in the T3 treatment, which yielded the best ES quality and yield. The substantial ES yield and quality in the T3 treatment can be attributed, at least in part, to the involvement of Chloroflexi in carbon and nitrogen cycles and the versatile capabilities of Gammaproteobacteria, such as utilizing various organic compounds and producing diverse secondary metabolites and antibiotics (Zarzycki et al., 2009; Sorokin et al., 2014; Bhattacharyya et al., 2021).

While our study provides rigorous data analysis on the impact of soil microorganisms on ES growth, it has certain limitations. To further substantiate our conclusions, exploring the transplantation of rhizosphere microbial communities could be valuable. This process entails the selection of specific microorganism types for introduction to the target crop. We would then evaluate changes in the crop’s performance and other physiological outcomes. This approach would yield a more in-depth understanding of the ecological processes governing microorganism-rhizosphere-crop interactions (Jiang et al., 2022; Jin et al., 2023).

## 5 Conclusion

**Figure 8:**
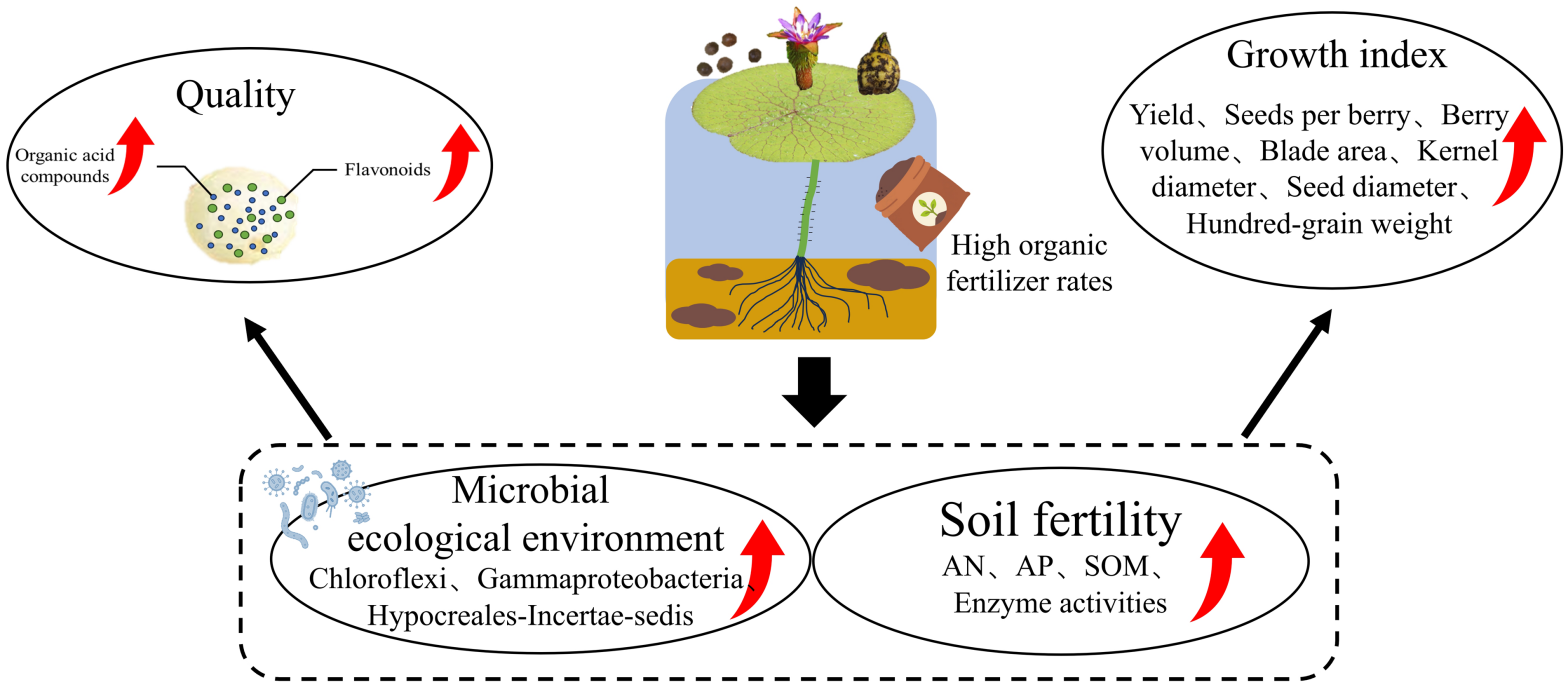
High-quantity organic fertilizers can enhance both the yield and quality of Euryales Semen in the Euryales Semen-wheat rotation system.

In this study, morphological characteristics and metabolome information were integrated to evaluate the effects of fertilizer regimes on ES yield and quality. The results suggest that implementing a 7:3 ratio of organic fertilizer to chemical fertilizer within the ES-wheat rotation system resulted in improvements in both the yield and quality of ES (Figure 8). This is primarily attributed to the improvement of soil properties and soil enzyme activity, the reshaping of the microbial ecosystem, and the increased abundance of microbial taxa (such as Chloroflexi, Gammaproteobacteria, and Hypocreales-Incertae-sedis). VPA showed that soil properties, enzyme activities, and their interactions cumulatively explained 90.80% of the variation in the ES yield and metabolome. These findings further revealed the internal mechanism of the influence of different fertilization methods on the yield and quality of ES through soil physical and chemical properties, soil enzyme activity, and soil microbial structure. This research aims to lay a theoretical foundation for developing high-yielding green ecological planting models for ES.

## Data availability statement

The data presented in the study are deposited in the Sequence Read Archive (SRA) repository (https://www.ncbi.nlm.nih.gov/), accession number PRJNA1025979.

## Author contributions

DL: Writing – original draft, Writing – review & editing, Data curation, Formal analysis, Investigation. CQ: Investigation, Methodology, Validation, Writing – original draft, Writing – review & editing. XC: Writing – original draft, Data curation, Formal analysis. YC: Writing – original draft, Formal analysis. HY: Writing – review & editing, Conceptualization, Supervision. QW: Writing – review & editing, Conceptualization, Funding acquisition, Supervision.

## Footnotes

^1^ mona.fiehnlab.ucdavis.edu/downloads

^2^ https://www.arb-silva.de/documentation/release138/

^3^ https://www.omicstudio.co.uk

^4^ https://www.omicstudio.cn
