# Supplementary material for: Effect of different fertilization strategies on the yield, quality of Euryales Semen and soil microbial community
Source: Front Microbiol. 2023 Nov 30;14:1310366. doi: 10.3389/fmicb.2023.1310366 (PMC10719947; doi:10.3389/fmicb.2023.1310366)
Supplement: Supplementary file 1 [file Data_Sheet_1.docx]

**Supplementary Material**

**Table S1** Primer nucleotide sequence for PCR amplification of the V3-V4 region of the bacterial 16S rRNA gene and the internal transcription interval 2 (ITS2) region of the fungal ribosomal RNA gene.

| Name | Region | Sequence (5ʹ–3ʹ) |
| --- | --- | --- |
| 16S | 341F | CCTACGGGNGGCWGCAG |
|  | 805R | GACTACHVGGGTATCTAATCC |
| ITS2 | ITS1FI2 | GTGARTCATCGAATCTTTG |
|  | ITS2 | TCCTCCGCTTATTGATATGC |


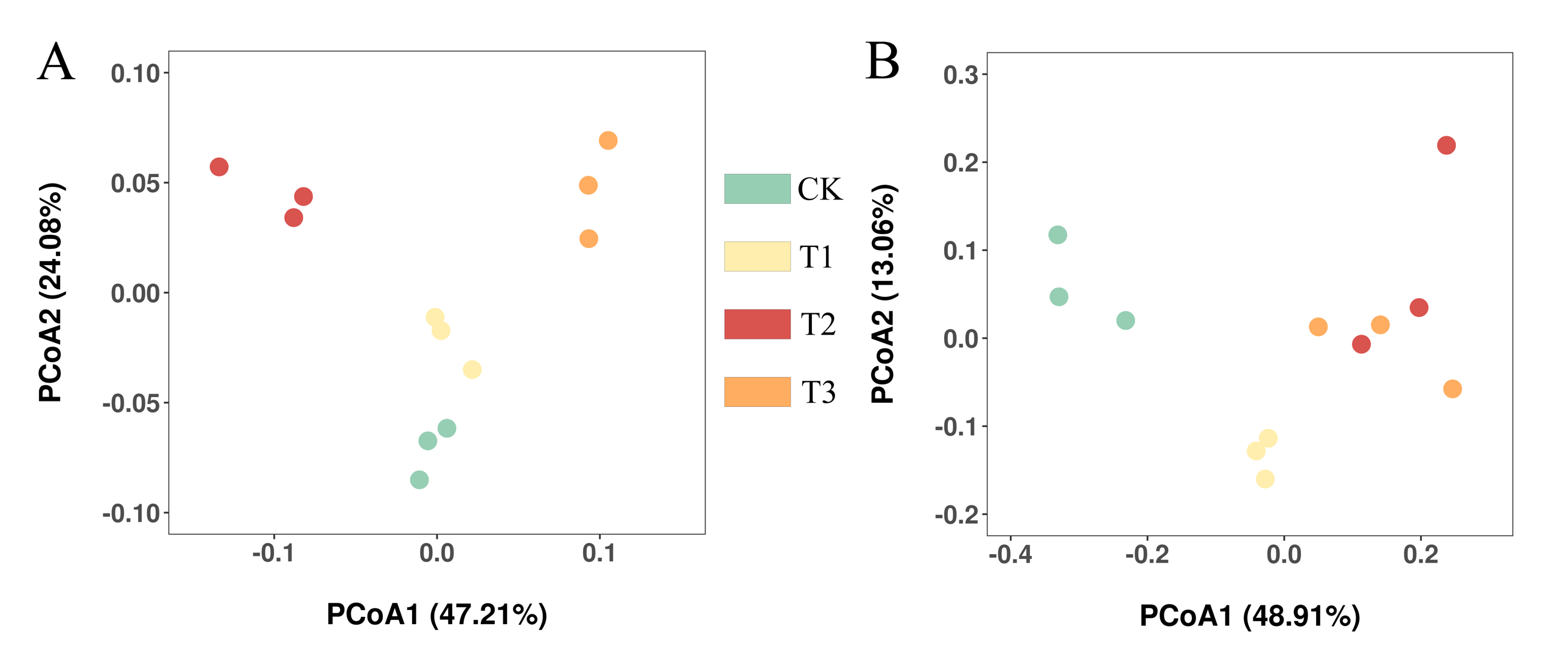


**Figure S1** Principal co-ordinate analysis of the community structure of bacteria (A) and fungi (B) under different fertiliser treatments.


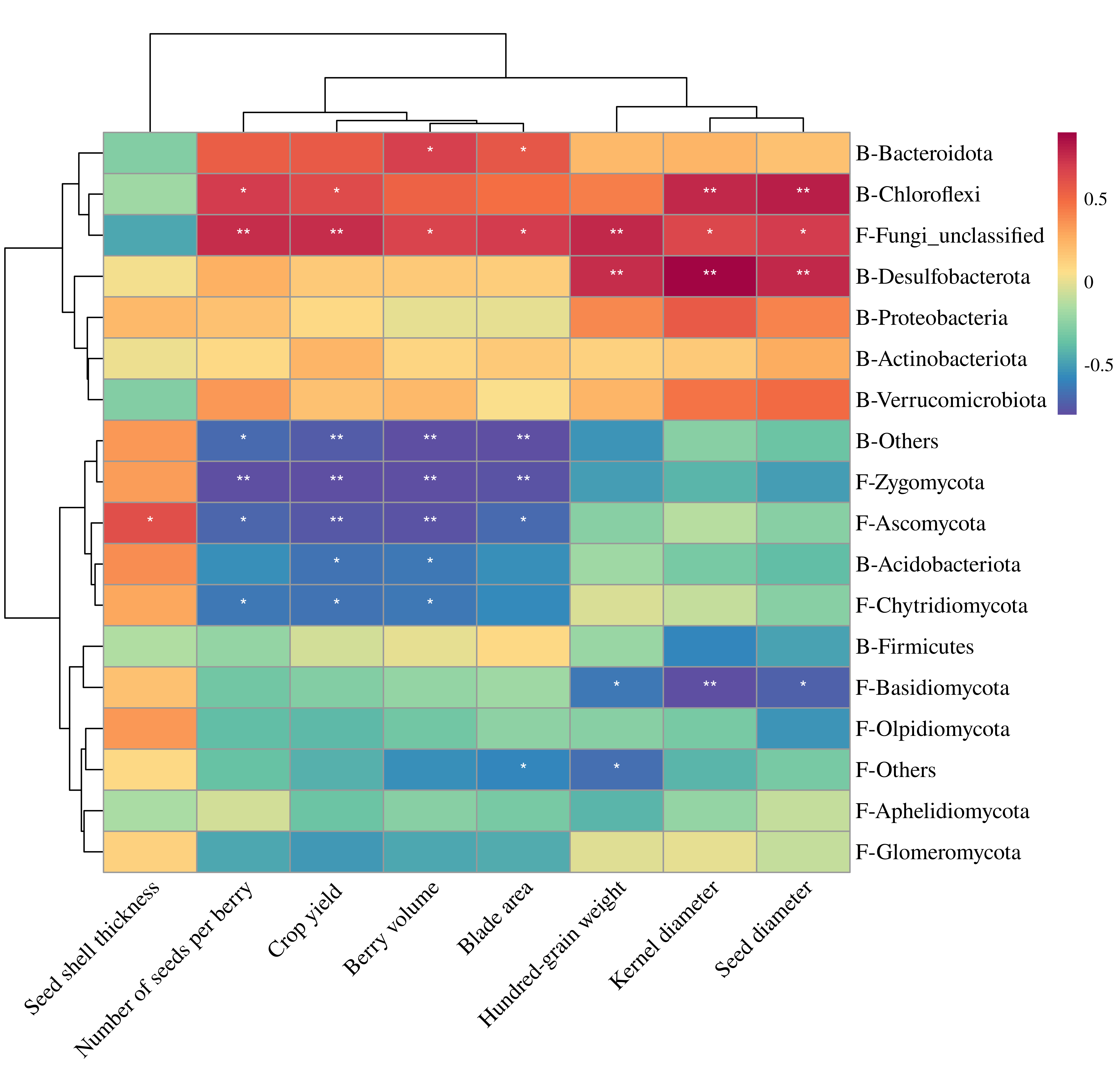


**Figure S2** Correlation network between agronomic traits and microorganisms (Top 8 bacterial and fungal abundance) between different treatment groups. * *P* < 0.05, ** *P* < 0.01.
